# ‘THA for DDH: replacement principles and techniques – femoral side’

**DOI:** 10.1530/EOR-2026-0069

**Published:** 2026-05-01

**Authors:** Konstantinos Konstantinidis, Dimitrios Grammatikopoulos, Eustathios Kenanidis, Alexander Maslaris, Pedro Dantas, Eleftherios Tsiridis

**Affiliations:** ^1^ Academic Orthopaedic Department, Papageorgiou General Hospital, School of Medicine, Faculty of Health Sciences, Aristotle University of Thessaloniki, Thessaloniki, Greece; ^2^ ICAROS Orthopaedic Center, Thessaloniki, Greece; ^3^ Department of Orthopaedic Surgery, Hospital CUF Descobertas, Lisbon, Portugal; ^4^ NOVA Medical School, Lisbon, Portugal

**Keywords:** DDH, developmental dysplasia of the hip, dysplastic hips, total hip arthroplasty, Crowe classification, Hartofilakidis classification, femoral stems, subtrochanteric osteotomy

## Abstract

- Developmental dysplasia of the hip (DDH) remains a leading cause of early hip osteoarthritis and poses considerable technical challenges during total hip arthroplasty.
- Existing classification systems focus on the abnormal relationship between the femoral head and the acetabulum without emphasising the femoral morphological variations, which are equally significant in surgical planning.
- Dysplastic femora often demonstrate excessive neck anteversion, variable neck–shaft angles, reduced offset, and metaphyseal–diaphyseal mismatch with narrow intramedullary canals. These anatomical variations often compromise the abductor mechanism and alter hip biomechanics.
- Thorough preoperative planning is crucial and should include the evaluation of leg-length discrepancy, reconstruction of the hip centre of rotation, assessment of femoral version and canal morphology, implant selection, and consideration of femoral shortening osteotomy. Computed tomography-based planning can enhance preoperative planning in complex cases.
- Both cemented and cementless stems align with positive findings in DDH patients. Cemented stems enable reliable version control, decrease intraoperative fracture risk, and may be beneficial for patients with poor bone quality. In mild cases, most uncemented stems are suitable. More severe deformities necessitate specialised implant designs with conical fluted stems, which provide strong fixation and excellent long-term survivorship. Modular and custom-made options also achieve favourable outcomes in high-grade deformities.
- Femoral shortening osteotomies are often needed to restore biomechanics, correct abnormal version, and prevent nerve injury during reduction. The subtrochanteric osteotomy has been linked with excellent results in treating severe DDH.
- High-quality research is essential to deepen our understanding of proximal femur morphological abnormalities and to enhance surgical results.

## Introduction

Developmental dysplasia of the hip (DDH) is one of the most common skeletal deformities, with prevalence rates that vary globally (1, 2). Hip dysplasia involves a combination of morphological abnormalities affecting both the acetabulum and the femur. Undetected hip dysplasia is a leading cause of premature hip osteoarthritis (2, 3, 4). Total hip arthroplasty (THA) for DDH remains a technically demanding procedure that requires a thorough understanding of anatomical abnormalities and proficiency in complex techniques (5). Traditionally, detection and assessment of these deformities have focused more on the acetabulum or its relationship with the femoral head, with comparatively less attention paid to anatomical abnormalities of the femoral side (4). Limited data are available regarding femoral head morphology, head–neck geometry, proximal femoral version, and femoral canal dimensions and shape in dysplastic hips, including whether these femoral features vary with the severity of dysplasia. These underreported deformities and technical difficulties continue to render THA in DDH patients a challenging task for orthopaedic surgeons (5). The aim of this paper is to review current concepts of femoral reconstruction in THA, including principles and techniques, for dysplastic hips.

## Classification systems

Several classification systems have been developed to describe the anatomical features of hip dysplasia and guide surgical treatment (6). These classifications detail the severity and pattern of acetabular deficiency, changes in the femoral shape, or both. However, most of them mainly focus on the relationship between the femoral head and the acetabulum to define dysplasia, without a detailed analysis of the specific morphological features of either the acetabulum or the femur. Among these, the systems described by Crowe *et al.* and Hartofilakidis *et al.* remain the most widely adopted (7, 8). The Crowe classification assesses the vertical displacement of the femoral head relative to the acetabulum, while the Hartofilakidis system classifies dysplastic hips based on the position of the femoral head inside or outside the acetabulum, with an emphasis on acetabular anatomy. Additional classification systems have been developed to improve or adapt traditional ones; however, they also largely overlook emphasising the unique anatomic characteristics of the proximal femur (9, 10, 11, 12, 13, 14, 15).

## Femoral challenges in DDH

Understanding proximal femoral anatomy is crucial for selecting the best surgical approach, fixation technique, and femoral stem in patients with DDH (5, 16). However, the proximal femoral shape in patients with hip dysplasia remains poorly documented. Although several small studies have studied the unique anatomical features of the dysplastic proximal femur, few are comparative in design, and most involve relatively small patient groups. Variations in femoral morphology can occur, including femoral version, neck–shaft angle, femoral offset, femoral canal shape, and the position of the greater and lesser trochanter (16, 17, 18, 19, 20, 21, 22, 23, 24).

### Femoral neck version

In most studies, the majority of patients with DDH show increased femoral neck anteversion (18, 19, 20, 21, 22, 24). However, it remains uncertain whether neck anteversion consistently increases in proportion with the severity of dysplasia or how often retroversion may occur instead (19, 24). Sugano *et al.* reported that patients with DDH showed an average increase in femoral neck anteversion of 10–14° and that 23% of dysplastic hips had anteversion exceeding 40°, compared with only 7% in age-matched controls (20). Noble *et al.* found that femoral anteversion increased by 5–16° in dysplastic hips, with the magnitude of the increase rising with deformity severity (21). In a radiologic study assessing 247 dysplastic hips using computed tomography (CT), Argenson *et al.* reported increased proximal femoral anteversion, ranging from 1 to 80°, with high individual variability (22). In another CT study, 50 symptomatic dysplastic hips were evaluated, with a mean femoral anteversion of 19 ± 12° (24). Although 52% showed excessive femoral neck anteversion, retroversion was also observed in eight hips. Obviously, the widespread use of CT preoperatively may reveal the prevalence of retroversion in dysplastic hips.

### Neck–shaft angle and offset

Whether the neck–shaft angle in dysplastic hips differs from that in normal hips remains uncertain. Patients with DDH exhibit more unusual neck–shaft angle values, including coxa vara and coxa valga, than normal individuals (17, 22, 23, 24, 25, 26). Boese *et al.* observed significant variability in reported angles and partly attributed this to inconsistent measurement methods (23). Increased femoral anteversion in dysplastic hips may also mimic coxa valga on anteroposterior views (27). Sugano *et al.* found that severe dysplasia was more strongly associated with a varus neck–shaft angle than with coxa valga (20). Another comparative study involving 47 dysplastic hips and 30 controls reported no significant difference in neck–shaft angle between Crowe I and II–III dysplasia and controls, but a smaller angle was observed in Crowe IV hips (25). Although it is also supported that the dysplastic hip is commonly associated with decreased femoral offset, evidence remains limited (17, 24). Wells *et al.*, in an analysis of 50 dysplastic hips, reported a mean neck–shaft angle of 136° ± 5 and noted that cam-type deformities and reduced head–neck offset are frequent findings in DDH (24).

### Femoral canal and metaphyseal–diaphyseal mismatch

Metaphyseal–diaphyseal size mismatch is common in dysplastic femora and often occurs alongside excessive femoral hypoplasia and the loss of the normal metaphyseal flare (16, 19). Quite often, highly dysplastic hips show a narrow, straight intramedullary canal (16, 20, 22, 25). Although evidence is sparse, it appears that dislocation severity in dysplasia may be inversely related to femoral canal deformity, with Hartofilakidis type C femoral canal frequently appearing relatively normal (20). Again, standard radiographs may not accurately estimate canal diameter; CT scans are more reliable (24). The AP view often overestimates canal diameter, while the lateral view may underestimate it due to rotational mismatch between the metaphysis and diaphysis, worsened by excessive femoral anteversion and positioning. Liu *et al.* found that Crowe IV dysplasia showed significant changes in both intramedullary and extramedullary measurements, especially narrowing of the medullary canal at the lesser trochanter, compared to Crowe I–III and normal hips (25).

### Greater trochanter variations and distorted hip biomechanics

Hip biomechanics are significantly altered in DDH (26, 28, 29). The greater trochanter is usually positioned more posteriorly, a feature closely associated with increased femoral anteversion, suggesting a proportional relationship between these parameters (16, 20, 26). Consequently, the length and function of the hip abductors are affected, and they may become wasted due to dysfunction, often resulting in a positive Trendelenburg sign. Muscle quality in chronically dislocated dysplastic hips is compromised, leading to soft-tissue shortening and functional insufficiency of the abductors, as well as the hip flexor and extensor musculature (26, 28, 29). The main biomechanical challenges during reconstruction include achieving the proper femoral version, restoring femoral offset, and correcting leg-length discrepancy (5, 30).

## Preoperative planning

Comprehensive preoperative planning for DDH should revolve around the following key points: assessing preoperative leg-length discrepancy (LLD), femoral version, offset and canal morphology, reconstructing the hip’s centre of rotation, ensuring primary stability, estimating the need for a femoral shortening or derotational osteotomy, and selecting the appropriate implants (5, 30, 31). Standard anteroposterior and lateral radiographs should be taken for templating. However, accurately measuring the femoral canal diameter on plain radiographs can be challenging. CT scanning from the hip to the knee may aid preoperative assessment by allowing precise evaluation of femoral canal morphology, femoral neck version, and supporting personalised prosthesis selection (5, 24, 30, 31). Conventional templating, whether manual or digital, is cost-effective but limited in reliability and reproducibility for predicting component size and position, especially in severe cases. Multiple studies suggest that CT-based 3D templating provides greater accuracy (32, 33). However, 3D planning has drawbacks, including increased planning time, increased radiation exposure, and higher costs. Preoperative planning should also include determining the surgical approach and assessing whether an osteotomy is necessary, as this may protect the sciatic nerve and restore abductor biomechanics (5, 30, 31). The standard posterior approach is considered the gold standard because it offers better access to the acetabulum and the femur, is extensile, and can address all types of deformities (34). Muscle-sparing mini posterior approaches have also been utilised in most Hartofilakidis 1–2 cases, yielding excellent results and significantly reducing complications (35). Recent literature also advocates the use of DAA in less severe cases; however, the evidence is limited, and exposure is challenging (34). Nonetheless, the choice of surgical approach remains at the surgeon’s discretion and experience. The selection of femoral stem type and fixation, as well as the need for femoral osteotomy, is extensively discussed in the subsequent sections of this review.

## Femoral reconstruction: general considerations

Following the acetabular reconstruction and positioning of the cup in the true acetabulum, the femoral reconstruction follows. The femoral head is cut, and if appropriate, 15–20 mm of the medial neck above the lesser trochanter is preserved to optimise proximal contact and stability of the femoral component. The femoral canal is identified and reamed to achieve diaphyseal contact. Based on the chosen type of stem, the femoral trial stem is inserted, and an attempt is made to reduce the hip. If reduction is unsuccessful, a complete capsulectomy is performed to improve exposure and allow for lengthening. This may involve releasing tendons, such as the iliopsoas and rectus femoris, from their bony attachments; an adductor tenotomy or a gluteus maximus tenotomy could also improve exposure. If the hip can be reduced, potential impingement sites and risks of dislocation are assessed. If the greater trochanter impinges against the posterior acetabular wall or the ischium, options include increasing the offset or performing a femoral rotational osteotomy. If the hip remains irreducible, a shortening femoral osteotomy is considered (36, 37, 38).

## Femoral management strategies

### Stem type and fixation method

The morphologic variability of the femur in patients with DDH presents unique challenges, especially when selecting the appropriate implant and fixation method for each case (5, 16, 38). Choosing the femoral stem design is critical, as each option offers specific advantages and disadvantages. Typically, the choice of implant is influenced by the degree of deformity (37). Although various stem types are widely used, most existing literature on implant outcomes consists of case series and single-arm cohort studies, with relatively few large-scale comparative studies between different stems. Furthermore, most relevant research is retrospective, involves small sample sizes with significant variability in DDH severity, and either assesses older implants or, when modern implants are used, lacks long-term follow-up.

Despite this relative paucity of high-quality comparative research, it is paramount to highlight the evidence from the existing literature to guide clinical practice and inform future studies. A summary of current comparative clinical studies between different femoral implants is depicted in Table 1 (39, 40, 41, 42, 43, 44, 45, 46, 47, 48, 49, 50, 51, 52, 53, 54, 55, 56).

**Table 1:** Summary of comparative clinical studies between different femoral implants.

Authors	Year, country	Study type	Crowe grade	Stem groups (number of hips)	Osteotomy	Time of surgery	Age* (y)	FU* (mo)	Key findings^†^
Choy *et al.* (39)	2013, Australia	RCS-R	N/A	Short Exeter (94), standard Exeter (234)	N/A	1999–2010	N/A	12–84	Short = Standard (SR)
Mutlu *et al.* (40)	2016, Turkey	RCS	III–IV	Corinium (43), SL-plus (43)	TSO	1999–2010	48	71	**Corinium** < SL-plus (DUR & NUR), NE on (HHS & MAP)
Ozden *et al.* (41)	2017, Turkey	RCS	IV	Synergy/Image (22), echelon (23)	S-C SO	2000–2014	41	120	**Echelon** > Synergy/image (SR); **Echelon** < Synergy/image (NUR, IOFR)
Koyano *et al.* (42)	2017, Japan	RCT	N/A	Super secur-fit (36), CentPillar (36)	N/A	2004–2007	52	110	= Radiologic outcomes
Kong *et al.* (43)	2019, China	RCS	I–II	S-ROM (100), LCU (96)	N/A	2015–2017	41	24	**S-ROM** < LCU (LLD); **S-ROM** > LCU (FJS)
Inoue *et al.* (44)	2021, Japan	RCS	IV	Cem (Exeter) (13), unc (S-ROM/K-MAX) (13)	V-S SO	2009–2017	69	61	**Cem** > Unc: (early function); **Unc** < Cem: (TU)
Di Martino *et al.* (45)	2021, Italy	RCS-R	N/A	M-anat (1072), NM-anat (363), M-con (1020), NM-con (1267), M-tap (548), NM-tap (1491)	N/A	2000–2017	60	104	= Across all groups (SR)
Kaneuji *et al.* (46)	2021, Japan	RCS	I–IV	Non-C-type (17), C-type (87)	N/A	1990–1999	44	295	**C-type** > Non-C-type (SR)
Liu *et al.* (47)	2022, China	RC-CS	I–IV	MS (542), M-DS (582), DS (66)	Unspecified osteotomy on 1.3%	1999–2019	50	N/A	M-DS > **MS** = **DS** (IOFR)
Kayaalp *et al.* (48)	2022, Turkey	RCS	II–III	SL-plus (17), synergy (17)	No	2012–2017	43	61	Synergy > **SL-plus** (SSh); Synergy = SL-plus (HHS)
Zha *et al.* (49)	2022, China	RCS	IV	Link ribbed (14), synergy (9), LCU (14)	DC SO	2009–2016	50	36	LCU > **Link Ribbed** = **Synergy** (TU, MUR, ALR)
Miyazaki *et al.* (50)	2023, Japan	RCS	IV	Unc M (14), cem PT (23)	TSO	1996–2018	62	105	Unc M > **Cem PT** (SSh, IOF)
Hüsken *et al.* (51)	2024, Netherlands	RCS-R	N/A	Cem (1458), unc (5477)	N/A	2007–2021	56	78	Cem = Unc (5 & 10 y SR)
Rai *et al.* (52)	2025, India	SR & MA	I–IV	Modular (292), monoblock (622)	Various	N/A	46	112	Modular = Monoblock (HHS, SR, IOF, NUR)
Jia *et al.* (53)	2025, China	RC-CS	I–IV	LCU (81), S-ROM (227), corail (26)	TSO	2010–2019	45	107	S-ROM > **LCU** = **Corail **(thigh pain)
Kitade *et al.* (54)	2025, Japan	RCS	N/A	FMS-anatomic (36), anatomic fit (83)	N/A	1998–2014	59	184	FMS-Anatomic = Anatomic fit (20 y SR)
Wang *et al.* (55)	2025, China	RCS	IV	S-ROM (38), Wagner (37)	TSO	2013–2021	N/A	36	S-ROM > **Wagner** (OT, BL); **S-ROM** > Wagner (HHS); **S-ROM** < Wagner (TU)
Albayrak *et al.* (56)	2025, Turkey	RCS	III–IV	Summit (39), SL-plus (31), Wagner (37)	TSO	2004–2014	45.8	173	**Wagner** > Summit = SL-plus (mHHS, SR, IOF)

* Mean values.

^†^ Studied outcomes in parentheses; stem with superior outcome is in bold; = similar, >higher, <lower.

RCS-R, retrospective cohort study – registry based; RCS, retrospective cohort study; RCT, randomised controlled trial; RC-CS, retrospective case–control study; SR & MA, systematic review and meta-analysis; N/A, not available; Cem, cemented stem; Unc, uncemented stem; M, modular; NM, non-modular; Anat, anatomic; Con, conical; Tap, tapered; non-C-type, non-circumferential porous coated stem; C-type, circumferential porous coated stem; MS, metaphyseal stem; M-DS, metaphyseal–diaphyseal stem; DS, diaphyseal stem; PT, polished tapered; TSO, transverse subtrochanteric osteotomy; S-C SO, step-cut subtrochanteric osteotomy; V-S SO, V-shaped subtrochanteric osteotomy; DC SO, double chevron subtrochanteric osteotomy; NE, no effect; SR, survival rate; DUR, delayed union rate; NUR, non-union rate; HHS, Harris hip score; MAP, Merle d’Aubigne–Postel scale; IOFR, intraoperative fracture rate; LLD, leg length discrepancy; FJS, forgotten joint score; TU, time to union; SSh, stress shielding; MUR, malunion rate; ALR, aseptic loosening rate; FU, follow-up; y, years; mo, months; OT, operative time; BL, blood loss; mHHS, modified Harris hip score.

#### Cemented stems

Cemented stems offer several advantages, including easier and more precise control of version and a reduced risk of intraoperative and postoperative periprosthetic fractures (32, 50, 57, 58). Cemented stems can better accommodate abnormal femoral anteversion and irregular intramedullary canal morphology than cementless stems. While cemented fixation can be used in any case, regardless of patient age or femoral deformity, it may be particularly beneficial for patients with compromised bone quality. Cemented stems can also be used successfully in severe dysplasias where a shortening and derotational subtrochanteric osteotomy is necessary. Although there are concerns about potential cement leakage into the osteotomy site, this can be easily prevented by impacting autograft from the femoral head onto the endosteal side of the osteotomy, as shown by Charity *et al.* (57).

**Figure 1:**
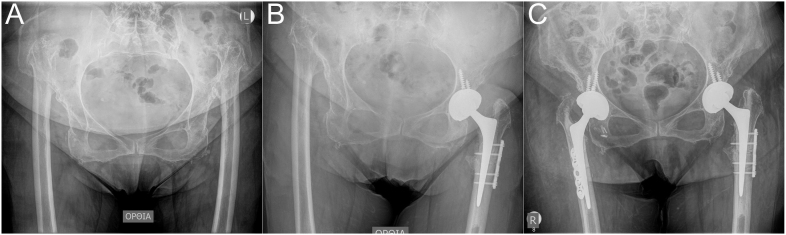
Preoperative (A) and postoperative (B and C) radiographs of bilateral Hartofilakidis C2/Crowe IV developmental dysplasia of the hip managed with hybrid total hip arthroplasty using cemented Exeter stems combined with transverse subtrochanteric osteotomy at two separate stages. The osteotomy site was stabilised using a dynamic compression plate and screws.

**Figure 2:**
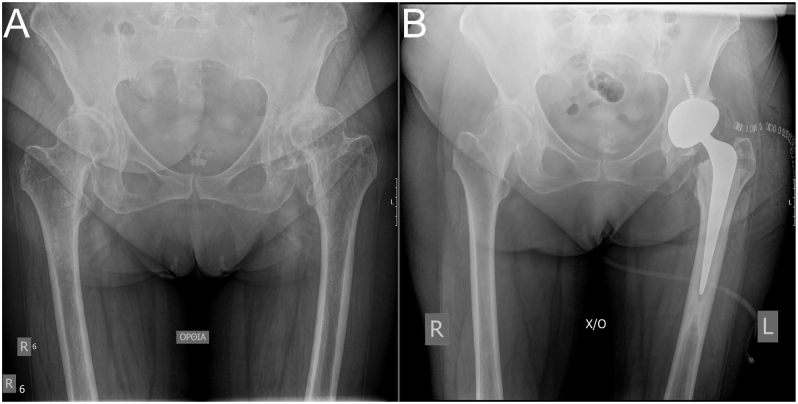
Preoperative (A) and immediate postoperative (B) radiographs of a Hartofilakidis B1 developmental dysplasia of the left hip treated with hybrid total hip arthroplasty using a cemented Exeter stem.

A variety of cemented stem options are available, most of which show low complication rates and good survivorship in mid- and long-term follow-up. A study by Miyamoto *et al.* with a mean follow-up of 10.7 years on DDH patients who underwent THA with a cemented stem demonstrated excellent survivorship, emphasising the long-term potential of cemented implants (58). In addition, comparative studies between short and standard-length Exeter stems implanted in DDH patients reported similar positive outcomes regardless of the implant type size (39). A study on 18 Crowe IV hips that underwent THA and transverse subtrochanteric osteotomy with the Exeter stem reported highly favourable outcomes (57). In a similar study by Huang *et al.* involving Crowe IV patients treated with THA and transverse subtrochanteric osteotomy, the use of the Lubinus SP II cemented femoral component yielded positive outcomes at an average follow-up of 49 months (59). A retrospective comparative cohort study of Crowe IV patients who underwent THA with subtrochanteric shortening osteotomy, using either cemented or cementless femoral component fixation, showed superior early clinical outcomes in the cemented stem group, while the cementless stem group demonstrated a quicker time to bone union at the osteotomy site (44) (Figs 1 and 2).

#### Cementless stems

Cementless stems are valued for their ability to promote osseointegration and their straightforward application (5, 36). In mild cases, most types of cementless stems are appropriate, while more severe deformities require specific implant designs, some of which have shown promising long-term results (30, 36, 37).

##### Tapered stems

**Figure 3:**
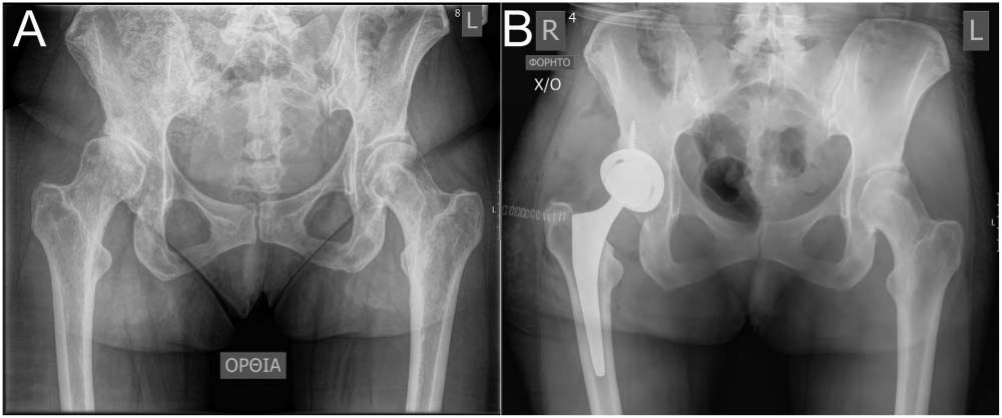
Preoperative (A) and immediate postoperative (B) radiographs of a Hartofilakidis A dysplastic right hip treated with cementless total hip arthroplasty using a tapered stem.

Tapered stems demonstrated favourable outcomes in many DDH studies (40, 46, 48, 49, 51). They are generally considered less suitable in dysplastic cases with excessive femoral neck anteversion. In cases of rotational mismatch between the metaphysis and diaphysis, conventional tapered stems may be undersized, leading to malalignment, malrotation, or femoral fracture (43). Although these implants show excellent survival in long-term follow-up studies, they are also linked to higher rates of subsidence and proximal stress shielding, which may increase the risk of periprosthetic fracture or aseptic loosening, although the actual impact on clinical outcomes remains uncertain (48, 50). A study of Crowe III–IV patients undergoing THA with a rectangular tapered stem reported successful mid-term outcomes at a mean follow-up of 41.6 months (60) (Fig. 3) (Table 1).

##### Cylindrical fully porous coated stems

These prostheses were traditionally used in DDH patients because their design allowed them to bypass proximal femoral abnormalities and provide diaphyseal fixation (61). Their application in DDH cases is now limited. Research reporting on long-term outcomes is relatively scarce, but one study assessing a cylindrical fully porous coated stem in DDH patients showed excellent survivorship at 12-year follow-up, despite some degree of stem subsidence in all implants (61).

##### Conical fluted stems (Wagner type)

Conical fluted diaphyseal-engaging (Wagner type) stems are widely used in dysplastic hips, facilitating easier adjustment of anteversion, often avoiding the need for derotational osteotomy, and providing strong rotational and vertical stability due to their conical taper and presence of flutes. They are particularly suitable for narrow femoral canals. Recent research suggests that these implants offer adequate stability after a subtrochanteric osteotomy, potentially eliminating the necessity for internal fixation of the osteotomy site (62, 63, 64). Multiple studies have shown excellent survivorship over very long-term follow-up periods, although intraoperative fractures and stem subsidence remain a potential issue. Nonetheless, Wagner-type stems consistently demonstrate comparable or superior outcomes relative to other types of cementless prostheses in comparative studies (Table 1).

Three retrospective cohort studies assessing the use of Wagner stems in DDH patients, reporting exceptional survival rates of 98.9, 80.5, and 97% at 15, 20, and 20 years of follow-up, respectively (62, 63, 64). Notably, the presence of radiological findings such as stem subsidence and radiolucent lines was not associated with worse clinical outcomes.

##### Modular stems

Modular prostheses are also common, providing a valuable option for managing abnormal anteversion and metaphyseal–diaphyseal mismatch, often avoiding the need for derotational osteotomy. By allowing the decoupling of the metaphyseal and diaphyseal fit, even in cases of excessive neck–shaft angles or straight intramedullary canals, modular stems enable the surgeon to adjust the femur’s length, offset, and version. The most prominent example of these stems is the S-ROM, which can deliver excellent angular and rotational stability. Downsides of these implants include high cost, reduced strength at the modular junctions, and potential interface issues such as fretting or corrosion (65).

A comparative study between Wagner Cone Stems and S-ROM stems on Crowe IV patients undergoing THA with subtrochanteric osteotomy revealed similar healing and safety profiles for both implants (55). In a retrospective study on modular stems used in the treatment of Crowe IV patients with THA combined with subtrochanteric osteotomy, the 10-year survivorship rate was remarkable, with zero component revisions. A recent meta-analysis of observational studies comparing outcomes between modular and monoblock stems in DDH patients undergoing THA with subtrochanteric osteotomy found similar functional results and mid- and long-term survival rates between the two types of implants (52) (Table 1).

##### Custom-made stems

**Figure 4:**
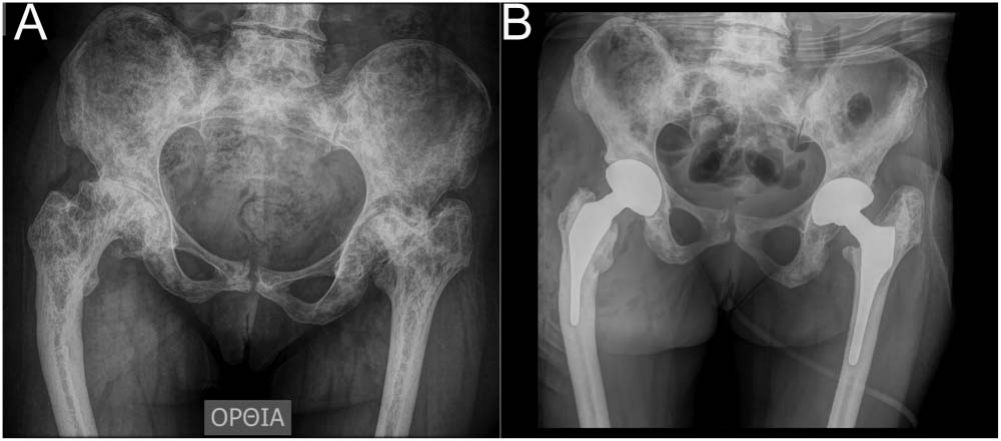
Preoperative (A) and immediate postoperative (B) radiographs of bilateral Hartofilakidis A dysplastic hips in a patient with sickle cell anaemia and abnormal proximal femoral anatomy, treated with hybrid total hip arthroplasty using custom-made stems at two separate stages.

Custom stems are mainly designed and manufactured for patients with abnormal proximal femoral sizes and shapes, offering a valuable alternative in more severe DDH cases, with encouraging results reported in the literature (66). Possible drawbacks include lengthy production times, high costs, and limited availability. Hitz *et al.* conducted a retrospective study of DDH patients of varying severity treated with THA using a custom-made stem without an osteotomy (66). The authors reported positive outcomes at a minimum follow-up of 10 years. Custom-made cemented stems are also available (Fig. 4).

##### Short stems

Short stems have been suggested as an alternative in mild dysplasia; however, their role remains limited, mainly because they cannot provide enough rotational stability or achieve the proper anteversion. Recent research has indicated potential uses for these implants; however, the evidence is still insufficient at this stage (67).

## Femoral osteotomies

### Aim

The main goal of an osteotomy is to restore leg length, the lever arm and function of the abductor mechanism, and femoral anteversion (68, 69, 70). A specific aim of shortening osteotomies is also to prevent sciatic nerve palsy (68, 69, 71). The risk of sciatic nerve injury increases when the cup is positioned in the true acetabulum, especially when limb-lengthening exceeds 4 cm, although recent studies suggest this traditional limit can be safely surpassed with careful intraoperative nerve monitoring (71). Furthermore, the higher incidence of nerve injuries during these surgeries may be more related to anatomical changes in the sciatic nerve in dysplastic hips than to limb-lengthening procedures (70, 71).

### Types

Although osteotomies can be carried out for all types of DDH, they are more often reserved for high-grade dysplastic hips, specifically types III–IV (5, 38, 68, 69). Osteotomies are classified based on the deformity being corrected (such as shortening or derotation), the anatomical site of the osteotomy (proximal, shaft, distal), and the technique used (transverse, oblique, double-chevron, and step-cut) (69). The extent of dysplasia and the specific anatomical abnormalities determine the appropriate type of osteotomy for each case. In Crowe II hips, a derotational osteotomy may be necessary if there is a significant version abnormality. For Crowe type III and especially Crowe type IV, both a shortening and a derotational osteotomy are often required (36, 38, 68, 69).

### Subtrochanteric osteotomy

Subtrochanteric osteotomy is by far the most used osteotomy method because it preserves the abductor mechanism and the proximal femoral bone (68, 69, 70, 72). Careful preoperative and intraoperative planning of the osteotomy position is essential. Key steps for conducting the subtrochanteric osteotomy are outlined based on our experience.

**Figure 5:**
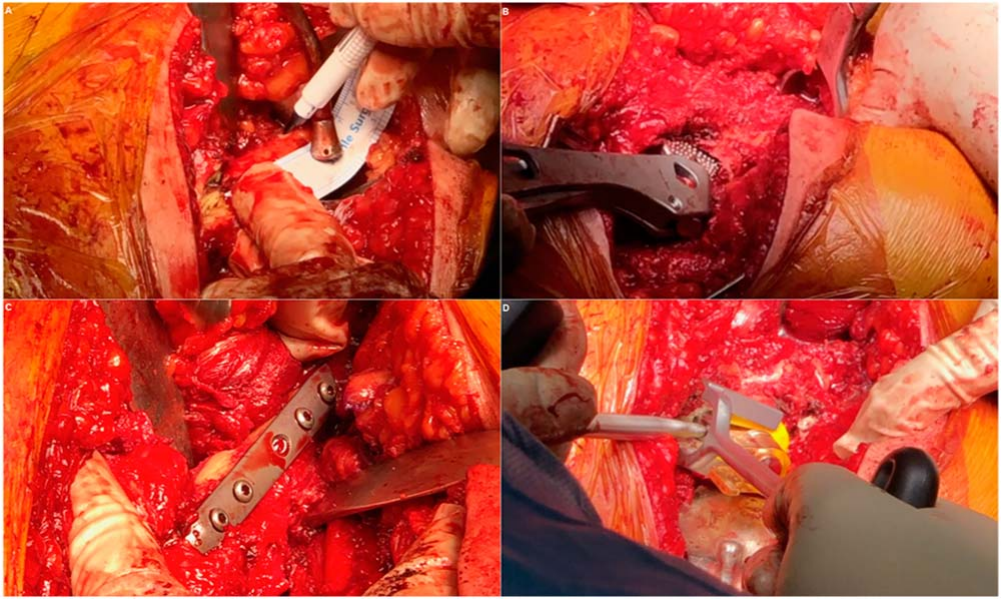
Transverse subtrochanteric osteotomy in a patient with Hartofilakidis C2/Crowe IV developmental dysplasia of the hip treated with cemented total hip arthroplasty. (A) Measurement of the length of the ensuing osteotomy. (B) The osteotomised femur is reduced, and a broach is inserted to perform a trial hip reduction. (C) Internal fixation of the osteotomy site on the posterior surface of the femur using a plate and screws. (D) Proximal pressurisation of the cement during final insertion of the stem. After polymerisation, extruded cement from the osteotomy site is removed with an osteotome, and the gap is filled with autograft.

After the cup is implanted in the true acetabulum, attention is then turned to the femur. If the lengthening is excessive and reduction is not possible, an osteotomy is ultimately necessary. Before shortening, reaming of the canal is initiated, and a rasp is inserted to check the length of the osteotomy site needed to achieve reduction without overstretching the sciatic nerve. The femur should be osteotomised below the metaphyseal flare of the stem, while also being proximal enough to allow the stem to achieve sufficient distal engagement to ensure stability at the osteotomy site. Typically, this osteotomy is performed about 10 cm distal to the tip of the greater trochanter in the subtrochanteric region. The bony segment is then excised, the broach is reinserted with the two bony ends reduced over each other, and trial reduction of the hip is performed. The position and version of the stem are checked to achieve leg length equality and stability. Then, the two osteotomised femoral ends are approached and fixed together with a 3.5 mm small fragment 5- or 7-hole dynamic compression plate (DCP) using screws at the back of the femur to facilitate stem insertion. The small gaps are filled with bone graft inside and outside the osteotomy. Subsequently, either a cemented or a cementless stem is inserted. For a cemented stem, a third-generation cementing technique is employed during stem insertion. The cement is not prevented from extruding outside the osteotomy site; it is only after setting that it is detached from the bony ends using an osteotome, and the gap is again filled with autograft (57, 70) (Fig. 5).

### Osteosynthesis methods

Several osteosynthesis methods for the subtrochanteric osteotomy have been described, including plate fixation, cables, cerclage wiring, and intercalary autograft application, while satisfactory healing rates have also been reported without fixation in cases of conical fluted stems. The most widespread and proven technique involves fixation of the osteotomy site with a 5- or 7-hole small-fragment DCP plate and 2 or 3 3.5 mm screws on each side of the osteotomy (57). This method, implemented in the study by Charity *et al.*, has yielded excellent outcomes, providing rapid and safe healing with minimal non-union rates (57). Sener *et al.* used a proximal diaphyseal step-cut shortening osteotomy stabilised with two to three cerclage wires and bone grafting in 28 DDH hips, with favourable outcomes at 4-year follow-up (73). Masson *et al.* performed a transverse subtrochanteric osteotomy and fixed it with a double-tension-band wiring technique in 31 Crowe III–IV hips, reporting low complication rates at a 12-year follow-up (74).

### Comparison of different techniques for subtrochanteric osteotomy

Transverse osteotomy is the most straightforward and frequently used technique (72, 75, 76). While it may potentially cause a mismatch between the proximal and distal segments and offer less inherent rotational stability, these issues can usually be mitigated with proper preoperative planning and internal fixation of the osteotomy site (57, 77). Conversely, oblique, step-cut, and double chevron osteotomies are considered more complex and provide less intraoperative flexibility regarding version and length correction. Although they are believed to have greater intrinsic stability than transverse osteotomies, a biomechanical study demonstrated comparable stability across all osteotomy techniques (76). In addition, a retrospective comparative cohort study analysed the complication rates of two osteotomy techniques in Crowe III–IV patients, namely, step-cut osteotomy and transverse osteotomy – showing a significantly higher complication rate in the first group (78). The excellent outcomes achieved by this method are highlighted in a recent systematic review and meta-analysis on THA with subtrochanteric osteotomy for severe DDH (72). Transverse osteotomy was the predominant technique among the included studies, while it was also linked to a lower rate of delayed union or non-union compared to other methods after subgroup analysis.

### Other osteotomies

Greater trochanter osteotomies are now rarely performed. They include the standard greater trochanter osteotomy, the trochanteric slide osteotomy, the trochanteric advancement osteotomy, and the extended trochanteric osteotomy. They provide excellent exposure of the femur and acetabulum, assist in lowering the femur, protect the abductor mechanism, and restore its level arm. However, they are associated with a relatively high non-union rate at the osteotomy site (69). Distal osteotomies are quite rare, primarily performed when there is a severe concomitant valgus knee deformity. In a study on Crowe type IV patients, outcomes between subtrochanteric shortening osteotomy and trochanteric slide osteotomy were retrospectively compared (79). Subtrochanteric shortening osteotomy was associated with better functional outcomes and lower rates of non-union.

### Complications

An osteotomy requires careful preoperative planning to avoid complications. Due to the complex nature of the procedure, including femoral dysmorphia and altered anatomical relationships between structures, multiple complications may occur, including malunion or non-union at the osteotomy site, intraoperative fracture, neurovascular injury, muscle weakness, heterotopic ossification, and hardware-related issues (72, 75). The risk of these adverse effects depends on the anatomical site and osteotomy technique, the fixation method used at the osteotomy site, and the stem type and implantation technique (cemented or cementless). However, a recent systematic review of 53 comparative studies on high-grade DDH examined outcomes across different osteotomy techniques and found no significant differences in complications, including non-union rates, between osteotomy types (75). The overall non-union rate ranged from 0 to 2%, while the overall aseptic loosening rate of the femoral component ranged from 0 to 7.14%. The highest infection rate (2.63%) was observed in oblique osteotomies.

## Conclusion

Managing the femoral side in dysplastic hips remains a significant challenge for surgeons. While outcomes are generally satisfactory, there is a need to further reduce complications, including LLD, abductor insufficiency, and gait disturbances. Conducting anatomical studies to understand the morphological features of the proximal femur in DDH is essential to identify differences from normal anatomy. This knowledge will improve preoperative planning and aid in selecting appropriate implants.

## ICMJE Statement of Interest

The authors declare that there is no conflict of interest that could be perceived as prejudicing the impartiality of the work reported.

## Funding Statement

This research did not receive any specific grant from funding agencies in the public, commercial, or not-for-profit sectors.

## Author contribution statement

The authors have full control of primary data and agree to allow the journal to review these data upon request.
